# Perioperative Outcomes of Using Different Temperature Management Strategies on Pediatric Patients Undergoing Aortic Arch Surgery: A Single-Center, 8-Year Study

**DOI:** 10.3389/fped.2018.00356

**Published:** 2018-11-27

**Authors:** Yuanyuan Tong, Jinping Liu, Lihua Zou, Zhengyi Feng, Chun Zhou, Ruoning Lv, Yu Jin

**Affiliations:** ^1^Department of Cardiopulmonary Bypass, Chinese Academy of Medical Sciences and Peking Union Medical College Fuwai Hospital, Beijing, China; ^2^Department of Anesthesiology, Beijing Tiantan Hospital, Capital Medical University, Beijing, China

**Keywords:** cardiopulmonary bypass, pediatric, lower body circulatory arrest, regional low-flow perfusion, aortic

## Abstract

**Background:** With the widespread application of regional low-flow perfusion (RLFP), development of surgical techniques, and shortened circulatory arrest time, deep hypothermia is indispensable for organ protection. Clinicians have begun to increase the temperature to reduce hypothermia-related adverse outcomes. The aim of this study was to evaluate the safety and efficacy of elevated temperatures during aortic arch surgery with lower body circulatory arrest (LBCA) combined with RLFP.

**Methods:** We retrospectively analyzed data from 207 consecutive pediatric patients who underwent aortic arch repair with LBCA & RLFP between January 2010 and July 2017 and evaluated different hypothermia management strategies. The overall cohort was divided into three groups: deep hypothermia (DH, 20.0–25.0°C), moderate hypothermia (MoH, 25.1–30.0°C) and mild hypothermia (MH, 30.1–34.0°C).

**Results:** The percentage of AKI-1 occurrences was significantly increased in the MH group (51.52%) compared to those in the DH (25.40%) and MoH (37.84%) groups (*P* = 0.036); prolonged hospital stay occurrences were decreased with elevated temperature (DH 47.62%, MoH 28.83%, MH 18.18%, *P* = 0.006). Neurological complications, peritoneal dialysis, hepatic dysfunction, 30-day hospital mortality, delay extubation occurrences were no significant among the groups. Logistic analysis showed that the MH group was negatively associated with post-op AKI-1 compared with the DH group [*OR* = 0.329 (0.137–0.788), *P* = 0.013], no differences were found between the MoH and the MH group. Compared to other groups, the intubation time (*P* = 0.006) and postoperative hospital stay (*P* = 0.009) were significantly decreased in the MH group. Multivariate logistic analysis showed hypothermia levels were not significant with prolonged hospital stay.

**Conclusions:** This retrospective analysis demonstrated that for pediatric patients undergoing surgeries with RLFP & LBCA, three different gradient temperature management strategies are available: deep, moderate, and mild hypothermia. Utilizing mild or moderate hypothermia is safe and feasible. Although the number of AKI-1 occurrences in the MH group was significantly increased compared to those in the other groups, further analysis showed no significance in the MoH and MH group, mild hypothermia management is as safe as others when used appropriately.

## Introduction

While hypothermia circulatory arrest (HCA) has historically been an essential technique for aortic arch repair, it is closely associated with variable hypothermia-associated complications such as cerebral vascular resistance increases with reduction in temperature (1), intracellular calcium overload disturbances and coagulation disorders (2, 3). Antegrade cerebral perfusion (ACP) is now available as an adjunct during HCA, which provides some measurement of uninterrupted cerebral perfusion during aortic arch repair. Clinical physicians have created a paradigm toward elevated temperature management to avoid potential complications while also reducing the cooling and rewarming time and speeding up the procedure. In pediatric aortic surgery, lower body circulatory arrest (LBCA) replaces the previous systemic circulatory arrest, and regional low-flow perfusion (RLFP) replaces the previous ACP. Therefore, for surgeries using LBCA and RLFP in combination, selection of the system temperature management strategy remains very controversial (4). Previous studies focused on adult populations have shown that improving temperature management strategies is feasible (5–7), and some studies reported favorable clinical results (8–12). However, relevant research on a pediatric population is lacking. A safe temperature management strategy for LBCA & RLFP in pediatric aortic surgery remains unclear, and the effectiveness of using this technique to protect distal organs remains to be determined.

To address this clinical problem, this study retrospectively evaluated the perioperative clinical outcomes of pediatric patients undergoing aortic arch surgery under the management of deep, moderate, and mild hypothermia with the aim of reviewing whether replacing deep hypothermia with mild or moderate hypothermia is feasible in the management of temperature during skilled surgical procedures.

## Patients and methods

### Study design and data extraction

The medical records of pediatric patients subjected to RLFP & LBCA during a procedure to repair an aortic obstruction with a concomitant intracardiac anomaly at the authors' institution during this period were reviewed. To make the study findings more comparable and meaningful, we focused on only pediatric biventricular patients. The specific inclusion criteria were as follows: one-stage aortic malformation correction under cardiopulmonary bypass (CPB) within 36 months of age; aortic malformation diagnosis included an interrupted aortic arch (IAA), isolated coarctation of aorta (iCoA), and aortic coarctation combined with hypoplastic aortic arch (CoA & HAA); concomitant lesions included simple lesions (such as a ventricular septal defect, atrial septal defect, patent ductus arteriosus (PDA), and patent foramen ovale), and complex lesions (such as aortic stenosis, pulmonary stenosis, mitral and tricuspid regurgitation and stenosis). Patients who underwent re-operative arch reconstruction, had a concomitant heart transplant, or had conversion from the thoracotomy approach to CPB support during the same operation were excluded.

Information regarding the patient demographics, perioperative risk factors, morbidities, intraoperative data, length of ICU stay, intubation time, postoperative hospital stay, 30-day mortality and incidence of adverse clinical outcomes was retrieved from a clinical database. The hypothermia grouping in this study was based on the lowest nasopharyngeal temperature, as LBCA & RLFP begin after the temperature drops to the lowest nasopharyngeal temperature. We categorized patients in our center into three groups according to the level of hypothermia used during the procedure: deep hypothermia [DH, 20.0 – 25.0°C, mean temperature 23.43 ± 1.29°C]; moderate hypothermia [MoH, 25.1 – 30.0°C, mean temperature 27.59 ± 1.58°C]; and mild hypothermia [MH, 30.1 – 34.0°C, mean temperature 31.86 ± 1.11°C].

Ethical approval for this single-center, retrospective cohort study (approval number: 2014-600) was provided by Fuwai Hospital. This study waived consent from their guardians because it is retrospective research.

### Intraoperative management

#### Anesthesia and surgical technique

Aortic surgery requires the placement of both radial and femoral arterial blood pressure monitors. After induction, the anesthesiologist places a jugular bulb catheter and near-infrared spectroscopy (NIRS) sensors to assess cerebral perfusion (guaranteed intraoperative level within 80% of the baseline level). At the same time, another NIRS probe is placed on the kidney to monitor renal oxygen saturation. Like the surgical procedures described in a previous study (13), the same RLFP & LBCA surgical procedures were used for all patients during distal and transverse aorta repair. All of these surgeries were performed by fixed surgical team.

#### Cardiopulmonary bypass management

During the period of RLFP & LBCA, circulation was halted in the lower body (spinal cord, abdominal viscera and double lower limb). RLFP has two different perfusion forms. During the cerebral-myocardial perfusion form of RLFP, surgeons first operated on the aortic malformation and then perfused the cardioplegia fluid and repaired the intracardiac malformation as the temperature increased. The other form of RLFP involved perfusing the cardioplegia fluid before correcting the aortic malformation. After the aortic correction was completed, the intracardiac deformity was repaired during the rewarming process (14). Temperature monitoring probes were placed in the nasopharynx and rectum. Different hypothermia management was based on clinical protocol and experiences in our center, it was determined both by surgeons and perfusionists. The patient's age, surgical complexity, type of surgery, expected circulatory time and patient's pathophysiological status played important roles to the decision. Generally, the nasopharyngeal temperature in cerebral-myocardial perfusion cases was approximately 30–32°C to ensure the beating of heart. When the temperature dropped to the proposed preset value, RLFP was performed at a flow rate of 40.26 ± 16.88 ml/ (kg. min). The flow rate and pressure were adjusted by both the right radial artery blood pressure and NIRS monitoring. α-stat acid-base management was used during surgery. During the regional perfusion period, the temperature was no longer dropping. Hematocrit was maintained between 0.21 and 0.27, and the colloid osmotic pressure (COP) was maintained between 12 and 16 mmHg and adjusted to the pathophysiology of each individual. After correction, when the nasopharyngeal temperature increased above 35°C, the circulation in the child was relatively stable, the heart rate was normal, and no obvious bleeding was observable, the CPB was gradually terminated.

### Clinical outcomes

The primary outcomes were a series of complications. Information regarding neurological complications (NCs) and peritoneal dialysis were defined according to hospital records, and NCs were defined as dystonia, coma, convulsions, etc. Postoperative hepatic dysfunction was defined as a postoperative bilirubin level >2.5 mg/dl or an increase in aspartate aminotransferase (AST) and alanine aminotransferase (ALT) levels to more than twice the baseline level. Delayed extubation was defined as mechanical ventilation support time ≥ 48 h. Prolonged hospital stay was defined as postoperative hospital stay ≥14 days. Postoperative acute kidney injury (AKI) was diagnosed according to the Kidney Disease: Improving Global Outcomes (KDIGO) criterion, which is described below.

**Table:** 

**Stage**	**Serum creatinine (SCr)**	**Urine output**
1	Increase to 1.5–1.9 times the baseline value, OR increase ≥0.3 mg/dl (≥26.5 mmol/l)	< 0.5 ml/kg per h for 6–12 h
2	Increase to 2–2.9 times the baseline value	< 0.5 ml/kg per h for ≥12 h
3	Increase >3 times the baseline value, OR SCr ≥4 mg/dl (≥353.6 mmol/l), OR Initiation of renal replacement therapy, OR eGFR < 35 ml/min per 1.73 m^2^ (< 18 years)	< 0.3 ml/kg per h for ≥24 h, ORAnuria for ≥12 h

The time frames for the serum creatinine increases were as follows:

- Increase in SCr ≥0.3 mg/dl (≥26.5 mmol/l) within 48 h
- Increase in SCr >1.5 times the baseline value within the prior 7 days

eGFR: estimated glomerular filtration rate; h: hours.

The secondary outcomes included the duration of intubation, ICU stay, length of postoperative hospitalization and anesthesia recovery times and the postoperative 30-day mortality (death within 30 days after surgery).

### Statistical analysis

Categorical variables are presented as frequencies and compared with the χ^2^-test or Fisher's exact test when appropriate. Continuous variables are presented as the mean and standard deviation (SD) or as the median interquartile range (range from the 25th to the 75th percentile, IQR), and Student's independent *t*-test or the Kruskal-Wallis H test was used for group comparisons. Binary logistic regression analysis was used to estimate the association between variables and stage 1 AKI (AKI-1) occurrences and prolonged hospital stays occurrences. Variables with *P* < 0.10 in univariate logistic regression analysis were subsequently subjected to multivariate logistic regression analysis. Statistical significance was determined by *P* < 0.05. Statistical analyses were performed using Statistical Package for Social Sciences (SPSS 23.0) software (SPSS Inc., Chicago, IL, United States).

## Results

### Patient characteristics

Of the 254 patients presenting with aortic malformations between January 2010 and July 2017, 47 patients did not meet the inclusion criteria and were thus excluded. A total of 207 patients were enrolled in the study and retrospectively analyzed.

The preoperative characteristics and demographic variables of the overall population and their distributions among groups are summarized in Table 1. No significant differences were observed in most of the baseline characteristics. Risk Adjustment for Congenital Heart Surgery (RACHS) was used to determine the surgery complexity (15), and the RACHS level 3 distribution was higher in the MH group than in the DH and MoH groups (*P* = 0.046). For aortic diagnosis, the lower temperature group had more IAAs (*P* = 0.001) and fewer iCoAs (*P* = 0.029).

**Table 1:** Baseline characteristics.

**Variables**	**Deep hypothermia (*n* = 63)**	**Moderate hypothermia (*n* = 111)**	**Mild hypothermia (*n* = 33)**	***P-*value**
Males	41 (65.08)	67 (60.36)	23 (69.70)	0.583
Age (month)	5.00 [3.00,12.00]	4.00 [2.00,9.00]	5.50 [3.00,8.50]	0.335
Neonates ^a^ proportion	5 (7.94)	16 (14.41)	2 (6.06)	0.242
Weight (kg)	5.60 [4.50,7.40]	5.5 [4.2,7.5]	6.4 [4.85,7.8]	0.534
Height (meter)	0.62 [0.56, 0.71]	0.62 [0.56, 0.68]	0.64 [0.59. 0.67]	0.329
BSA (m^2^)	0.30 [0.26,0.38]	0.30 [0.24,0.36]	0.32 [0.27,0.37]	0.303
EF (%, median)	65.00 [62.25,73.15]	65.00 [62.20,73.00]	65.00 [60.00,70.00]	0.632
**RACHS SCORING**				
3	40 (63.49)	69 (62.16)	28 (84.85)	0.046
4	23 (36.51)	42 (37.84)	5 (15.15)	0.046
**AORTIC DIAGNOSIS**				
IAA	26 (41.27)	29 (26.13)	3 (9.09)	0.001
iCoA	32 (50.79)	66 (59.46)	26 (78.79)	0.029
CoA & HAA	5 (7.94)	16 (14.41)	4 (12.12)	0.431
**ASSOCIATED LESIONS**				
VSD	50 (79.37)	89 (80.18)	25 (75.76)	0.859
ASD	12 (19.05)	21 (18.92)	4 (12.12)	0.642
PDA	32 (50.79)	56 (50.45)	9 (27.27)	0.049
Mitral valve insufficiency	13 (20.63)	18 (16.22)	6 (18.18)	0.764
Tricuspid valve insufficiency	7 (11.11)	15 (13.51)	3 (9.09)	0.754
Patent foramen ovale	13 (20.63)	31 (27.93)	5 (15.15)	0.252
Mitral valve stenosis	2 (3.17)	1 (0.90)	1 (3.03)	0.500
Aortic valve stenosis	2 (3.17)	4 (3.60)	0 (0.00)	0.483

*Values are number (%); median, [IQR] or mean ± standard deviation unless otherwise indicated*.

a *Neonates was defined as age ≤ 28 days at operation. BSA, body surface area; EF, ejection fraction; RACHS, Risk Adjustment for Congenital Heart Surgery-1 score; IAA, interrupted aortic arch; CoA, aortic coarctation; HAA, Hypoplastic aortic arch; VSD, ventricular septal defect; ASD, atrial septal defect; PDA patent ductus arteriosus*.

### Operative data and outcomes

In the MH group, the CPB duration, myocardial ischemic duration and LBCA duration were significantly shorter than those in the other groups (*P* < 0.05), but the regional perfusion flow was higher (*P* < 0.05). In addition, cerebral-myocardial perfusion was used in the MH and MoH groups, but there were no cases of cerebral-myocardial perfusion in the DH group (Table 2).

**Table 2:** Details during RLFP and LBCA.

**Variables**	**Deep hypothermia (*n* = 63)**	**Moderate hypothermia (*n* = 111)**	**Mild hypothermia (*n* = 33)**	***P*-value**
CPB duration (min)	122.00 [110.00,141.00]	112.00 [93.00,127.00]	83.00 [75.00,102.50]	0.000
Myocardial ischemic duration (min)	74.00 [64.00,85.00]	67.00 [44.00,85.00]	28.00 [21.50,38.00]	0.000
LBCA duration (min)	29.00 [25.00,38.00]	25.00 [16.00,32.00]	18.00 [14.00,24.00]	0.000
RLFP flow volume (ml/kg)	32.00 [25.00,38.69]	37.27 [31.76,45.29]	52.30 [41.83,62.18]	0.000
MAP (mmHg)	31.00 [30.00,39.00]	32.00 [30.00,37.00]	31.00 [30.00,36.5]	0.923
Nasopharyngeal temperature (°C)	23.43 ± 1.29	27.59 ± 1.58	31.86 ± 1.11	0.000
Rectal temperature (°C)	26.89 ± 1.92	30.19 ± 2.05	34.08 ± 1.28	0.000
Cerebral-myocardial perfusion (%)	0 (0.00)	27 (24.32)	31 (93.94)	0.000
Urine output during surgery (ml/kg)	4.00 [1.00,7.00]	4.17 [0.98,7.58]	5.00 [2.49,12.70]	0.101

*Values are number (%); median, [IQR] or mean ± standard deviation unless otherwise indicated. CPB, cardiopulmonary bypass; LBCA, lower body circulatory arrest; RLFP, regional low flow perfusion; MAP, mean artery pressure*.

### Clinical outcomes

In the entire cohort, the percentages of AKI-1 occurrences were 51.52% in the MH group, 37.84% in the MoH group and 25.40% in the DH group. The number of AKI-1 incidences was significantly higher in the elevated temperature group than in the other groups (*P* = 0.036). However, the incidences of stage 2 and stage 3 AKI and peritoneal dialysis were not statistically significant between the groups (*P* > 0.05). In addition, the overall 30-day mortality was 2.44% (*n* = 5), the differences between groups were not significant and the incidence of NCs, hepatic insufficiency and delayed extubation were not statistically significant between the groups (Table 3).

**Table 3:** Intra-hospital morbidities and recovery outcomes.

**Variables**	**Deep hypothermia (*n* = 63)**	**Moderate hypothermia (*n* = 111)**	**Mild hypothermia (*n* = 33)**	***P-*value**
**INTRA-HOSPITAL MORBIDITIES**				
NS complications (%)	1 (1.59)	0 (0.00)	0 (0.00)	0.303
AKI-1 (%)	16 (25.40)	42 (37.84)	17 (51.52)	0.036
AKI-2 (%)	16 (25.40)	18 (16.22)	4 (12.12)	0.194
AKI-3 (%)	3 (4.76)	2 (1.80)	1 (3.03)	0.548
Peritoneal dialysis (%)	1 (1.59)	1 (0.90)	1 (3.03)	0.698
Hepatic dysfunction (%)	29 (46.03)	57 (51.35)	16 (48.48)	0.793
30-day hospital mortality	2 (3.17)	1 (0.90)	2 (6.06)	0.237
Delayed extubation^a^	20 (31.75)	25 (22.52)	5 (15.15)	0.165
Prolonged Hospital stay^b^	30 (47.62)	32 (28.83)	6 (18.18)	0.006
**RECOVERY OUTCOMES**				
Intubation time(h)	27.00 [20.00,50.50]	25.00 [18.00,45.00]	18.00 [12.00,23.00]	0.006
ICU stay(h)	108.00 [49.00,163.00]	70.00 [42.00,139.50]	72.00 [25.25,112.25]	0.063
Post-op Hospital stay(h)	13.00 [10.00,18.00]	11.00 [7.00,14.00]	10.00 [7.00,12.50]	0.009
Anesthesia recovery time(h)	2.00 [1.25,3.50]	2.00 [1.00,3.00]	2.25 [1.75,3.20]	0.210

*Values are number (%) unless otherwise indicated. AKI, acute kidney injury; NS, nervous system. Post-op, postoperative*.

a *delayed extubation was defined as mechanical ventilation support time ≥ 48 h*.

b *Prolonged hospital stay was defined as postoperative hospital stay ≥14 days*.

The durations of postoperative anesthesia recovery time and ICU stay were not significantly different between the groups, whereas the durations of mechanical ventilation support (*P* = 0.006) and postoperative hospital stay (*P* = 0.009) significantly reduced as the temperature increased (Table 3).

### Regression analysis results

**Figure 1:**
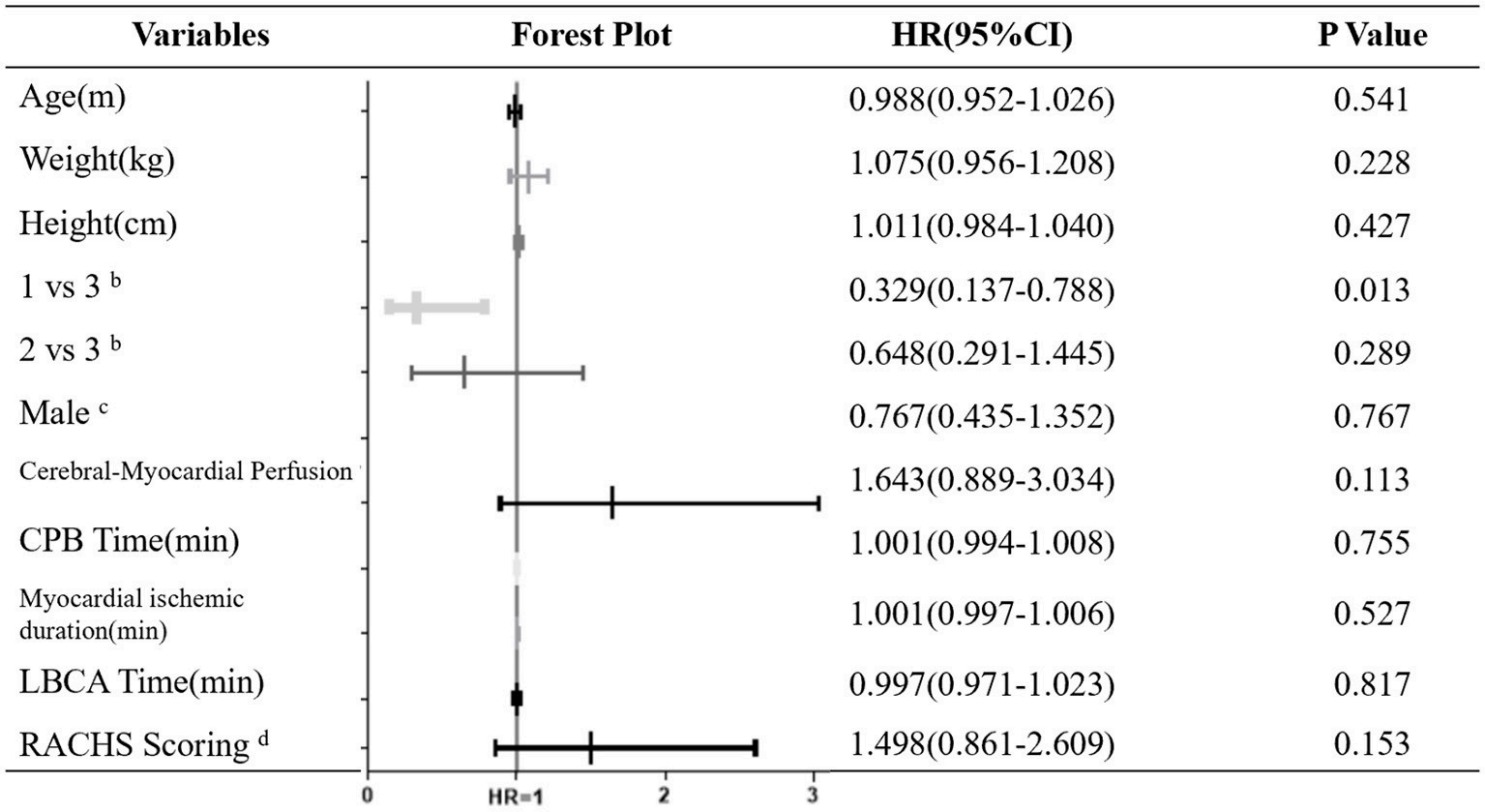
Univariate analysis and forest plot for Factors Associated with Postoperative AKI-1^a^. CPB, cardiopulmonary bypass; LBCA, lower body circulatory arrest; RACHS, Risk Adjustment for Congenital Heart Surgery-1 score. ^a^AKI-1 was defined according to KDIGO criterion, calculated as serum creatinine increase to 1.5–1.9 times the baseline value, OR increase ≥0.3 mg/dl (≥26.5 mmol/l) or urine output < 0.5 ml/kg per h for 6–12 h; ^b^Categorical variables, 1 for deep hypothermia, 2 for moderate hypothermia and 3 for mild hypothermia; ^c^Categorical variables, defined as 1 or 0; ^d^Categorical variables, defined as 3 or 4.

Univariate logistic regression analysis showed that the different hypothermia levels (1 for DH, 2 for MoH and 3 for MH) were significantly correlated with postoperative AKI-1 (*P* = 0.026). To be exact, the hypothermia level was negative correlated with postoperative AKI-1 occurrence in the DH and MH group [*OR* = 0.329 (0.137–0.788), *P* = 0.013], and there were no differences in the MoH and MH groups (Figure 1). Uni- and multi-variate regression analysis were performed concerning prolonged hospital stay, and variables with *P* < 0.10 in the univariate analysis were subsequently entered into the multivariate analysis. As shown in Table 4, none of these factors were independently associated with prolonged hospital stay.

**Table 4:** Univariate and multivariate analysis for factors associated with prolonged hospital stay ^a^.

**Variables**	**Univariate analysis**			**Multivariate analysis**		
	**HR**	**95% CI**	***P*-value**	**HR**	**95% CI**	***P*-value**
Age (m)	0.889	0.833–0.949	0.000	0.892	0.794–1.002	0.055
Weight (kg)	0.734	0.625–0.861	0.000	0.969	0.666–1.409	0.868
Height (cm)	0.935	0.902–0.969	0.000	1.000	0.920–1.088	0.992
1 vs. 3^b^	4.091	1.485–11.270	0.006	0.644	0.121–0.343	0.606
2 vs. 3^b^	1.823	0.687–4.834	0.228	0.327	0.071–1.501	0.150
Male^c^	1.328	0.720–2.449	0.363			
Cerebral-Myocardial perfusion^c^	0.237	0.105–0.536	0.001	0.290	0.072–1.168	0.082
CPB time (min)	1.023	1.013–1.034	0.000	1.014	1.000–1.029	0.056
Myocardial ischemic duration (min)	1.023	1.013–1.034	0.000	1.003	0.993–1.014	0.529
LBCA time (min)	1.046	1.016–1.076	0.002	1.008	0.971–1.046	0.681
RACHS scoring^d^	2.371	1.306–4.305	0.005	1.558	0.774–3.135	0.214

*CPB, cardiopulmonary bypass; LBCA, lower body circulatory arrest; RACHS, Risk Adjustment for Congenital Heart Surgery-1 score*.

a *Prolonged hospital stay was defined as postoperative hospital stay ≥14 days*.

b *Categorical variables, 1 for deep hypothermia, 2 for moderate hypothermia and 3 for mild hypothermia*.

c *Categorical variables, defined as 1 or 0*.

d *Categorical variables, defined as 3 or 4*.

## Discussion

A single-center retrospective cohort study on aortic arch surgery performed at the single center from January 2010 to July 2017 was conducted. We comprehensively assessed the safety and feasibility of using different systemic temperature management strategies on pediatric patients.

The one-stage repair of aortic arch congenital obstructions and concomitant intracardiac anomalies is now a routine but challenging procedure for pediatric patients. With the development of surgical skills, some clinical scholars have gradually increased the temperature during RLFP to reduce a series of complications caused by temperatures that are too low and appropriately shorten the time required for the entire operation. However, a recent study showed that adult aortic surgery patients subjected to direct deep hypothermic circulatory arrest (DHCA) showed improved long-term survival compared with patients not subjected to this technique (16), and elevated temperatures may increase the risk of ischemic organ injury. In our cohort, the duration of LBCA was 30.62 ± 8.99 min for the DH group, 25.78 ± 10.66 min for the MoH group, and 19.67 ± 8.74 min for the MH group; thus, the higher temperatures group had a shorter LBCA duration due to patient selection for mild hypothermia based on institutional surgical preferences. Compared with that reported by McCullough, the circulatory arrest time interval was much longer at the same hypothermia level in our study (17). This type of CPB management is due to advances in surgical technology, improved perioperative management and innovation in CPB strategies.

A previous study reported that innominate cannulation RLFP can provide additive circulation for lower infra-diaphragmatic organs and both lower extremities (18), but other studies did not report these benefits (19, 20). In aortic surgeries, clinicians have previously thought that congenital aortic obstruction may provide collateral circulation to the ischemic organs and therefore empirically increase the temperature during circulatory arrest. However, the tolerance of internal organs to different levels of hypothermia has not been clarified. In this study cohort, a higher number of temporary AKI-1 occurrences was observed in the MoH/MH group than in the DH group (*P* = 0.036). Logistic analysis showed that the MH group was negatively associated with post-op AKI-1 compared with the DH group [*OR* = 0.329 (0.137–0.788), *P* = 0.013], and no differences were found between the MoH and MH groups. This finding may due to a higher temperature management strategy during lower circulatory arrest. Hypothermia technology in CPB is utilized to reduce the metabolic requirements of important terminal organs. Low temperature achieves protection by reducing the metabolic rate, increasing intracellular pH, and increasing the content of high-energy phosphate. However, with the emergence of “tepid” RCP, the incidence of temporary renal insufficiency has increased. But, owing to the high ischemia tolerance of kidneys (kidneys can withstand ischemia for an hour at normal temperature, while exhibiting strong regenerative capability), a transient first-stage renal injury does not cause further damage (21). The strong tolerance of the kidney to ischemia has also been demonstrated in our study, showing a low incidence of renal dialysis in all groups (ranged 0.90–3.03%). Additionally, in-hospital observations also demonstrated that none of these transient kidney injuries had resulted in permanent damage. However, considering that postoperative AKI is an important indicator of patient rehabilitation and closely related to poor clinical prognosis (22), more prospective studies should be performed to determine the optimal clinical management temperature to further reduce the risk of postoperative AKI.

The incidences of NCs remain high even with the MoH & selective cerebral perfusion strategy, which is widely used today (23). It has been shown that autoregulation is intact during moderate hypothermia (25–32°C), and it is lost during deep hypothermia (18–22°C) (1). Additionally, research has shown that the hypothermia level is the only independent predictor of increased glial fibrillary acidic protein (GFAP, early marker of brain injury) (24) expression. Only one of the patients in this study reported severe NCs on the second postoperative day. Compared with other studies, the incidence of NCs was significantly lower in this cohort (25). We assumed that the low incidence (0.48%) of NCs might have been due to the routine use of NIRS (valuable noninvasive modality for cerebral oxygenation monitoring during cardiovascular surgery) real-time monitoring, pre-existing collateral blood circulation and the backflow of blood from the descending aorta to the spinal cord during RLFP. However, the most important reason underlying the discrepancy may be that we lacked an effective scoring system to evaluate early postoperative NCs (26).

Many studies on children undergoing congenital heart disease surgery have focused on postoperative recovery indicators to reduce family burden. Comprehensive has analysis demonstrated that these patients did not die due to MH or MoH management as opposed to DH management but rather because their conditions were more serious, and their operations were more complicated. Regarding the postoperative recovery variables, shorter mechanical ventilation support (*P* = 0.006) and postoperative hospital stay (*P* = 0.009) durations were observed in the MH group compared to those in the other groups. Regression analysis found different hypothermia level were not significant in prolonged hospital stay. These results further support that in specific diseases, MH and MoH management strategies are comparably safe.

## Limitations

This study does have two limitations. First, this study was not a randomized controlled trial, and the inherent bias associated with a retrospective study thus exists. Second, the limited data restricted us from reporting other potential effects of elevated temperatures. For example, postoperative behavioral scores were not analyzed to evaluate the possible NC complications in an early and timely manner.

## Conclusions

In summary, for pediatric patients undergoing surgeries with RLFP & LBCA, three different gradient temperature management strategies are available: deep, moderate, and mild hypothermia. Observations made with our cohort demonstrated that in specific diseases, under the management of a fixed perioperative team, we appropriately carried out a tepid regional low-flow perfusion management, and no significant adverse effects were observed. Moreover, moderate and mild hypothermia management is comparable in perioperative outcomes. More large-scale prospective studies are needed to further address this issue.

## Author contributions

YT and JL conceived the primary research question. LZ and ZF involved in the review conception and design. YT drafted the initial manuscript. JL provided interpretation and explanation of results and provided critical revision to the manuscript. CZ, RL, and YJ helped with language editing.

### Conflict of interest statement

The authors declare that the research was conducted in the absence of any commercial or financial relationships that could be construed as a potential conflict of interest.
